# Supplementary material for: A multiplexed, next generation sequencing platform for high-throughput detection of SARS-CoV-2
Source: Nat Commun. 2021 Mar 3;12:1405. doi: 10.1038/s41467-021-21653-y (PMC7930244; doi:10.1038/s41467-021-21653-y)
Supplement: Supplementary file 1 — Supplementary Information [file 41467_2021_21653_MOESM1_ESM.pdf]

A multiplexed, next generation sequencing platform for high-throughput detection of SARS-CoV-2

Marie-Ming Aynaud, J. Javier Hernandez, Seda Barutcu, Ulrich Braunschweig, Kin Chan, Joel D. Pearson, Daniel Trcka, Suzanna L. Prosser, Jaeyoun Kim, Miriam Barrios-Rodiles, Mark Jen, Siyuan Song, Jess Shen, Christine Bruce, Bryn Hazlett, Susan Poutanen, Liliana Attisano, Rod Bremner, Benjamin J. Blencowe, Tony Mazzulli, Hong Han, Laurence Pelletier, Jeffrey L. Wrana.

Supplementary information:

Supplementary Fig. 1 to 8

Supplementary Table 1 to 5

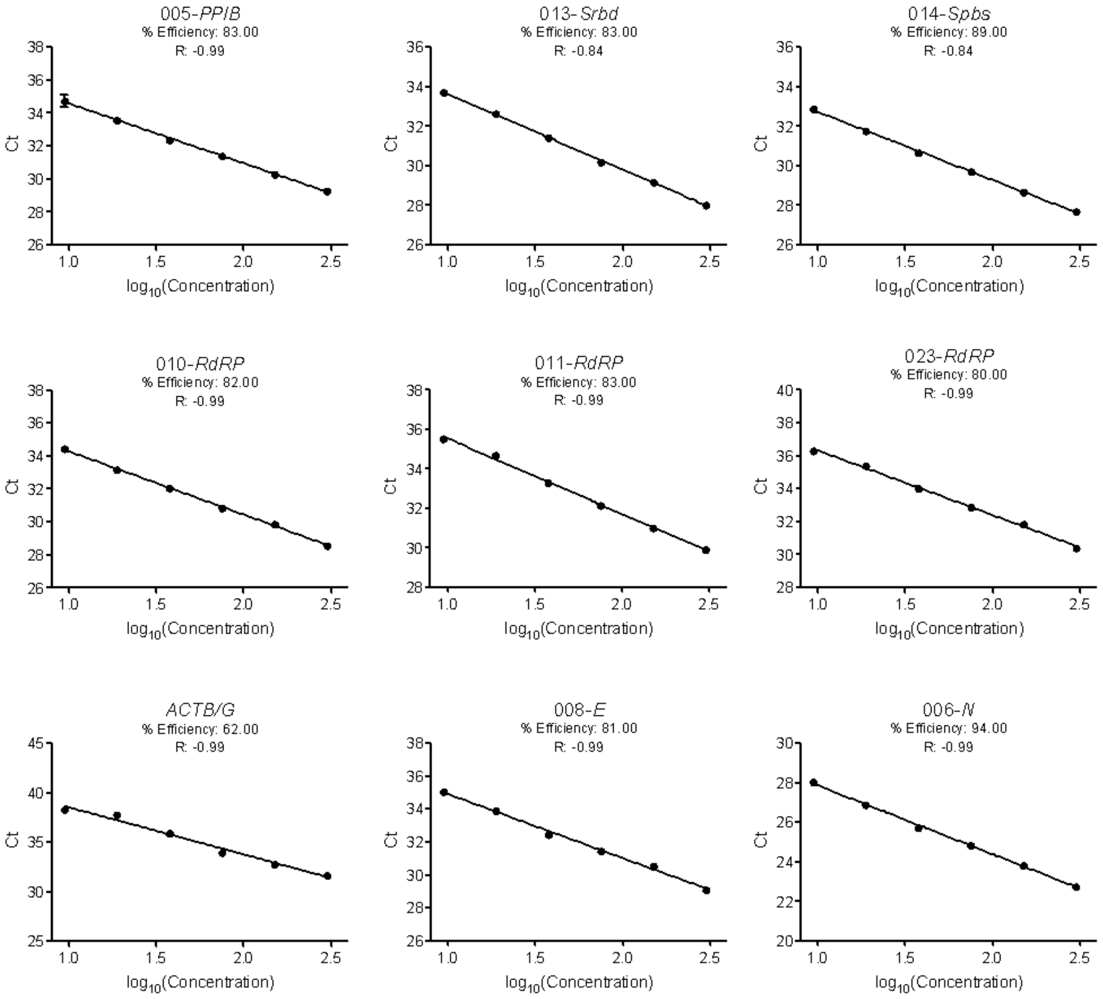

Supplementary Fig. 1: Efficiency of multiplex primers. Standard curve of Ct values (Y-axis) and log_{10}(Concentration) (X-axis) of 6 limited dilutions of SARS-CoV-2^{high} sample (LTRI-18) for 9 pairs of primers (see Supplementary Data 1). Each condition was tested in duplicate. Means are plotted for each point. The percent efficiency and the correlation (R) are calculated for each pair of primers after linear regression. Source data are provided as a Source Data file.

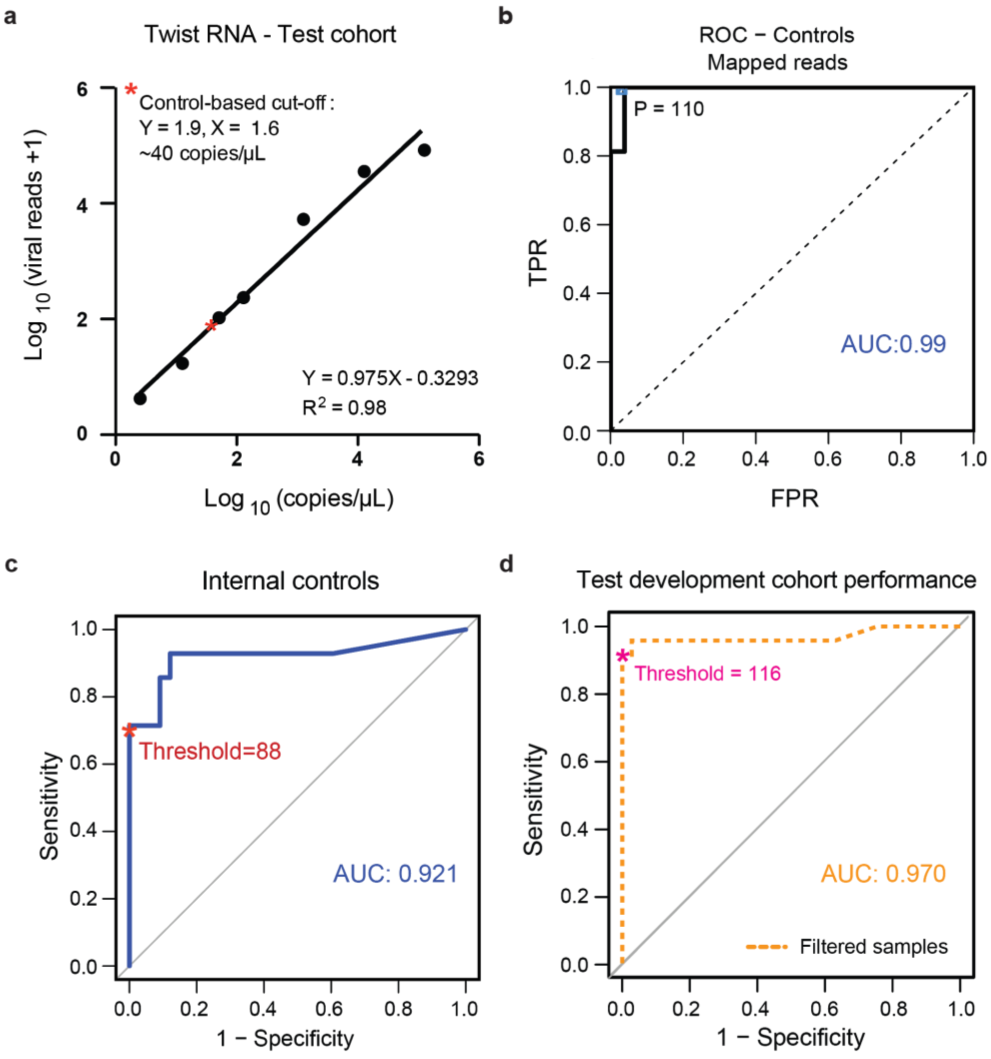

Cut-off selection

Optimal cut-off: 88 (PROC01)	Value	Lower Limit	Upper Limit
Sensitivity	0.714	0.419	0.916
Specificity	1	0.894	1

Supplementary Fig. 2: Using embedded controls as a training set for a control-based PR and ROC classifier. a Total viral read counts are plotted against estimated viral copies (copies/µL) obtained using synthetic Twist SARS-CoV-2 RNA with statistics indicated. The cut-off defined by PROC analysis (see panel **c**) is marked with a red asterisk. **b** Thresholding sample quality. coPR analysis on control samples: ROC of control samples for accurate detection of mapped reads are plotted. The optimal precision and recall read cut-off associated (P = 110) with the highest F1 (0.97) score, and AUC

(area under the curve) is indicated on the ROC plot. **c** Threshold for classification of positives in the test cohort. Total viral reads of negative (H2O and HEK293T) and positive (Twist dilutions) samples are used to calculate optimum cut-off by PROC and the defined threshold (P = 88) is plotted on the ROC curve. Values of sensitivity, and specificity at this cut-off are indicated (below). **d** Performance of C19-SPAR-Seq. ROC analysis on patient samples that passed RNA-QC threshold was performed using clinical diagnostic results (Seegene Allplex qRT-PCR assay, Supplementary Data 3) and total viral reads for patient samples (n = 112). AUC is indicated on the graph.

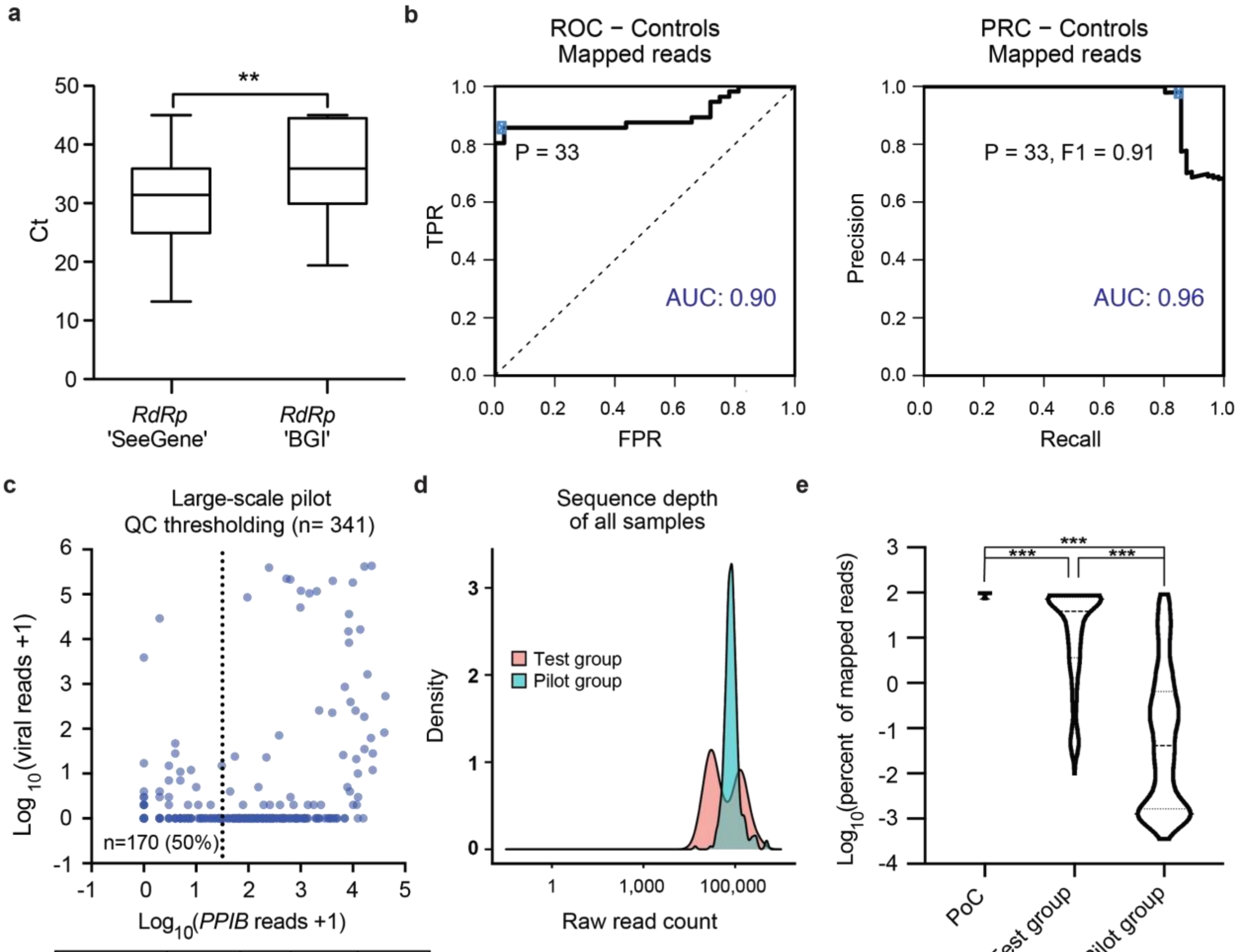

	Mean	Min.	Max.	Median
PPIB	1613	0	41874	29

Supplementary Fig. 3: Quality metrics assignment for the pilot cohort. a Comparison of Ct (*RdRP*) values in 'SeeGene' *versus* 'BGI' tests of the positive archival samples (Two tailed unpaired *t*-test, **: p = 0.003). Ct values are plotted for 'SeeGene' *versus* 'BGI' as a box and whisker (median + 95% confidence interval, and the maximum and minimum values). **b** coPR analysis on control samples. ROC and PRC of control samples are plotted and the optimal precision and recall cut-off (P = 33) associated with the highest F1 score (0.91) was calculated, as indicated in the PRC plot. **c** coPR thresholding of the pilot cohort. Plot of total viral reads +1 (Y-axis) *versus* *PPIB* reads +1 (X-axis) of 341 patient samples in a pilot cohort (see Methods) is shown with the threshold (*PPIB* read

counts > 33) to filter low-input samples marked. 170/341 (50%) samples were inconclusive (upper panel). Mean, minimum, maximum, and median values of *PPIB* and total viral read counts are indicated in the table (lower panel). **d** Sequencing depth of test development and pilot cohort. Distribution density of raw read counts for the test development (pink) and pilot (turquoise) cohorts are shown. **e** Read mapping percentages. Comparison of overall read mapping percentages between the PoC (Fig. 1), test (Fig. 2) and pilot cohort (n = 341). One way ANOVA - Tukey's multiple comparison test (***: adjusted p < 0.001).

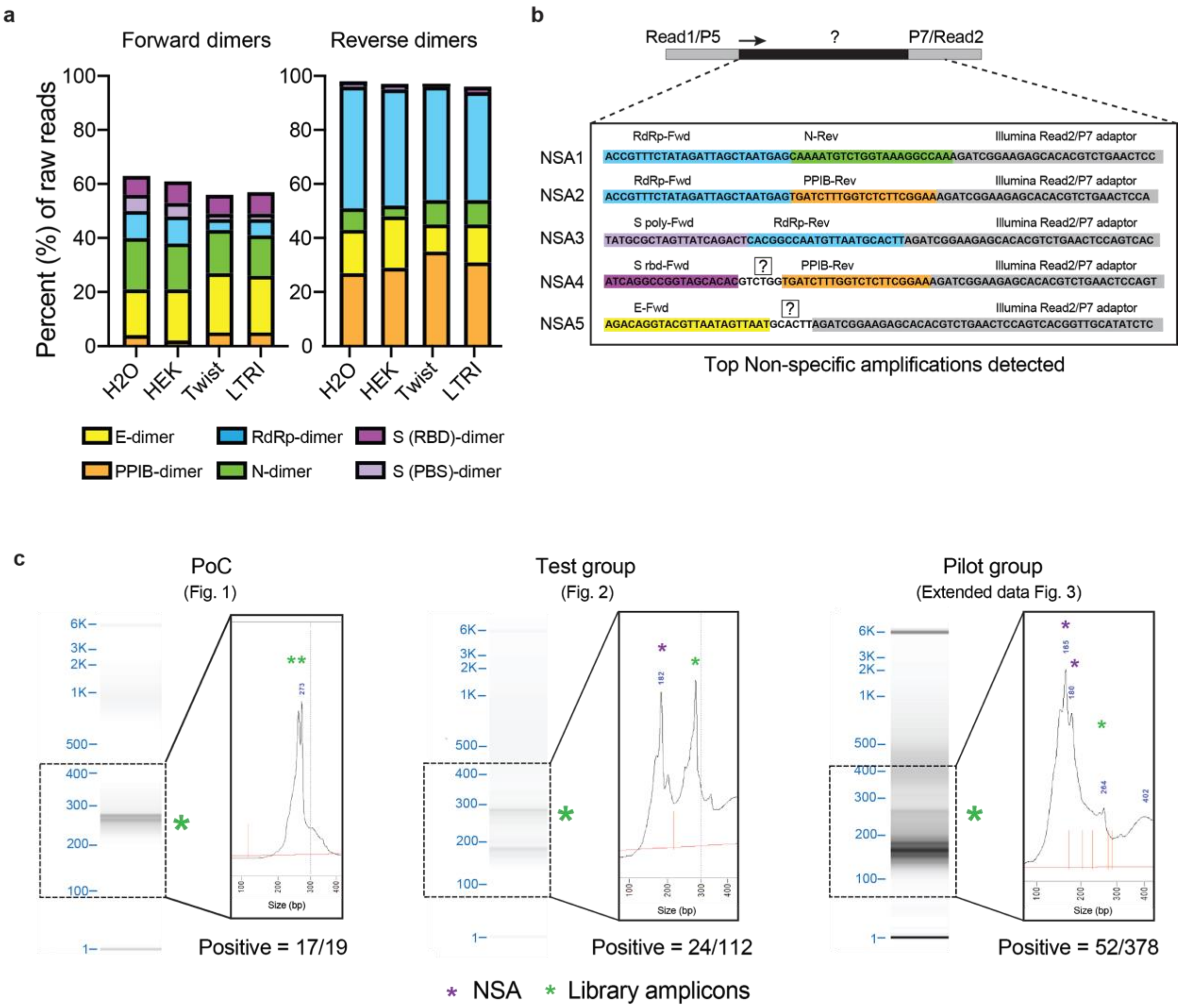

Supplementary Fig. 4: Non-specific amplification (NSA) in pilot cohort. a Analysis of NSAs in the pilot cohort. NSAs contaminating the C19-SPAR-Seq library were quantified and percentage of reads mapping to the indicated forward and reverse primers are plotted. **b** Schematic examples and sequences of the top 5 NSAs are shown. **c** Comparison of fragment analyzer profile of the PoC, test development, and pilot cohort libraries after 0.8X SPRI bead purification. Fragment separation (DNA gel) and blow-up view of the product abundance (electropherogram) are shown. Expected library

amplicons (green stars) and non-specific amplicons (red stars). Source data are provided as a Source Data file.

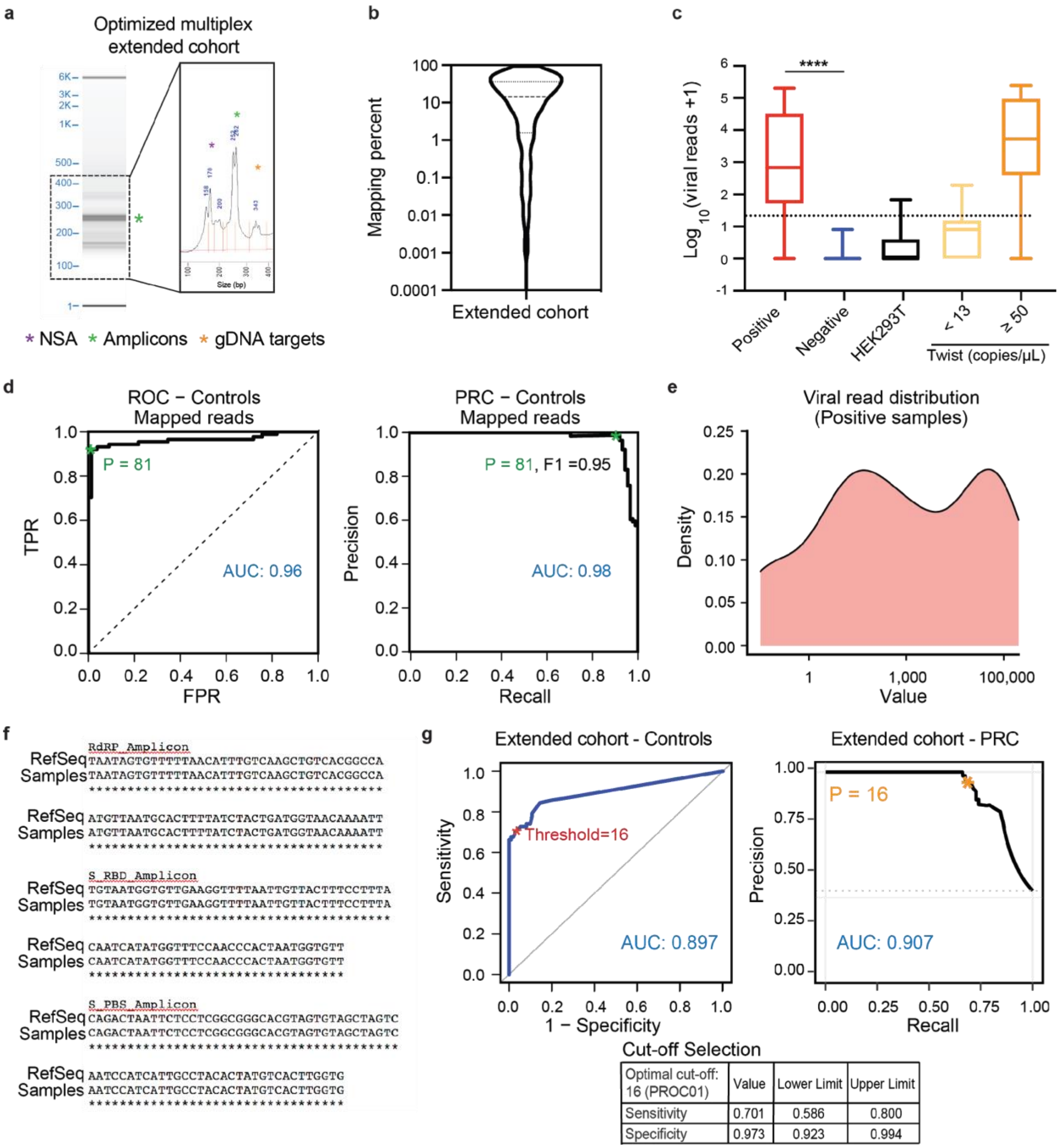

Optimal cut-off: 16 (PROC01)	Value	Lower Limit	Upper Limit
Sensitivity	0.701	0.586	0.800
Specificity	0.973	0.923	0.994

Supplementary Fig. 5: Suppressing non-specific amplicons and quality metrics assignment for the extended cohort. a Fragment analyzer profile of the extended cohort library using an optimized multiplex primer set targeting *ACTB/G*, *Spbs*, *Srbd*, and *RdRP*. Fragment separation (DNA gel) and blow-up view of the product abundance (electropherogram) is shown. Source data are provided as a Source Data file. **b** Mapping

percentage of the extended cohort. **c** Overall distribution of total viral reads in the indicated positive samples (n = 98, red), negative samples (n = 444, blue), HEK293T (n = 21, black), synthetic SARS-CoV-2-RNA (< 13.2 copies/µL, n = 6, yellow), and synthetic SARS-CoV-2-RNA (>= 50 copies/µL, n = 30, orange) are plotted as a box and whisker (median + 95% confidence interval, and the maximum and minimum values). Two tailed unpaired *t*-test of negative *versus* positive samples (****: $p = 6.84 \times 10^{-29}$). **d** coPR thresholding of sample quality and classification in the extended cohort. coPR analysis on control samples for sample quality yielded an optimal precision and recall read cut-off (P = 81) as indicated. **e** Distribution of $\log_{10}$ total reads +1 of the positive (n = 98) samples. **f** The dominant amplicon sequence from each patient sample is determined for each gene-specific primer and aligned to reference sequence. Representative alignment of the region between the gene specific primers are shown, where no variation was observed among the clinical samples processed. **g** Threshold for classification of the extended cohort. ROC on control samples (HEK293T and synthetic SARS-CoV-2 RNA control) was assessed to identify an optimal cut-off (P = 16) for classifying patient samples. Performance on the controls is summarized.

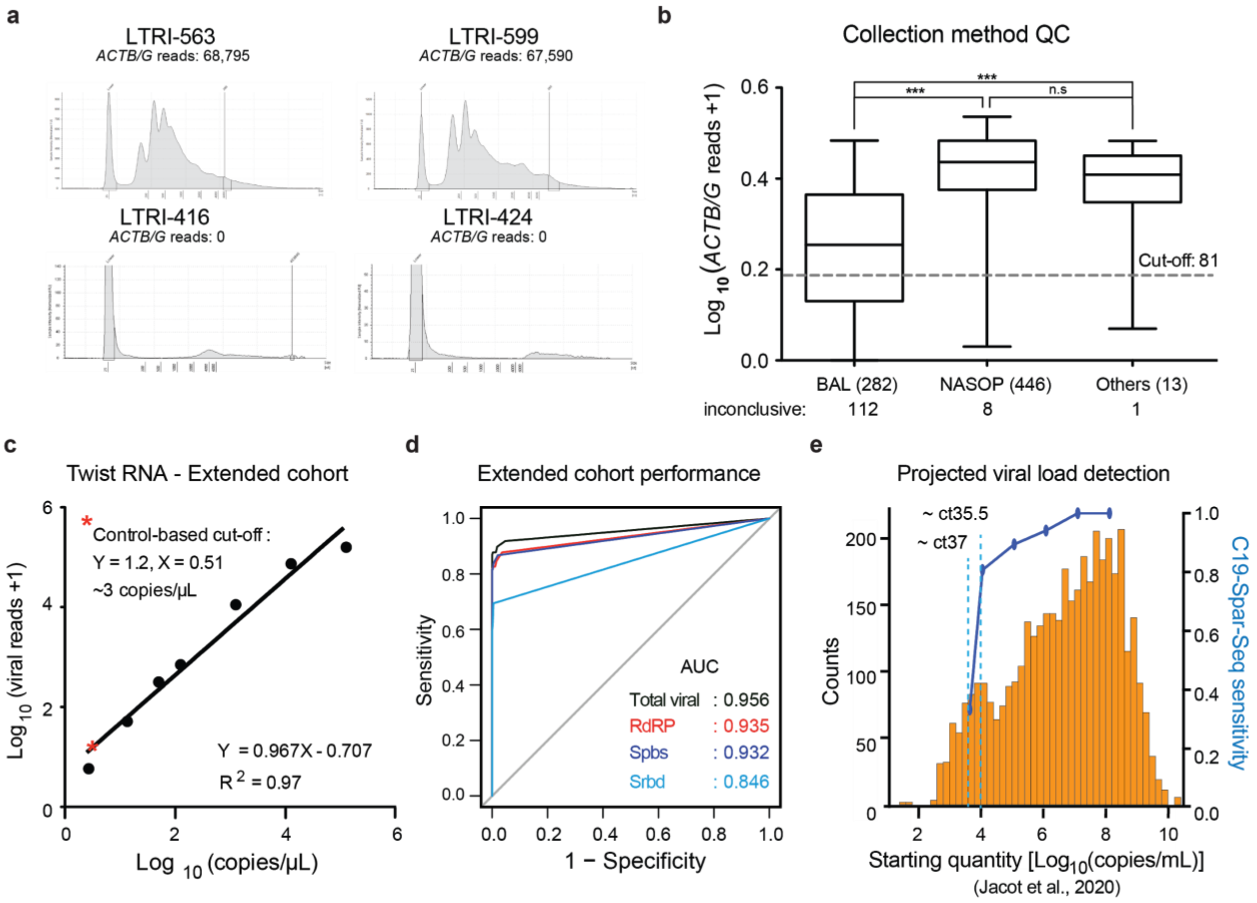

Minimum detection limit Log (viral load)	Projected C19-SPAR-Seq sensitivity
3.6 (~Ct 37)	0.92
4 (~Ct 35.5)	0.97

Supplementary Fig. 6: C19-SPAR-Seq performance. a RNA profile of BALs. RNA purified from ten BALs above and 10 below the QC threshold was profiled and two representative traces of each group are shown. *ACTB/G* reads are indicated for each sample. **b** *ACTB/G* reads according to collection type. *ACTB/G* reads are plotted for each collection type as a box and whisker (median + 95% confidence interval, and the maximum and minimum values). For each collection type group, the total number of samples are indicated in brackets (BAL, n = 282; NASOP, n = 446; Others, n = 13). The number of samples filtered by coPR (ACTB/G reads < 81) are indicated for each group. 1way ANOVA - Tukey's multiple comparison test (****: adjusted p < 0.0001, ns: non

significative) **c** Standard curve of total viral reads plotted against synthetic SARS-CoV-2 RNA concentrations obtained from C19-SPAR-Seq analysis of the extended cohort. **d** ROC curve analysis was performed for each of the indicated viral amplicons and the AUC is shown. **e** Projection of our C19-SPAR-Seq sensitivity onto the viral load data of ~4,000 patients from Jacot *et al.*, 2020 study[17]. Minimum detection limit and C19-SPAR-Seq sensitivity values are indicated in the table below.

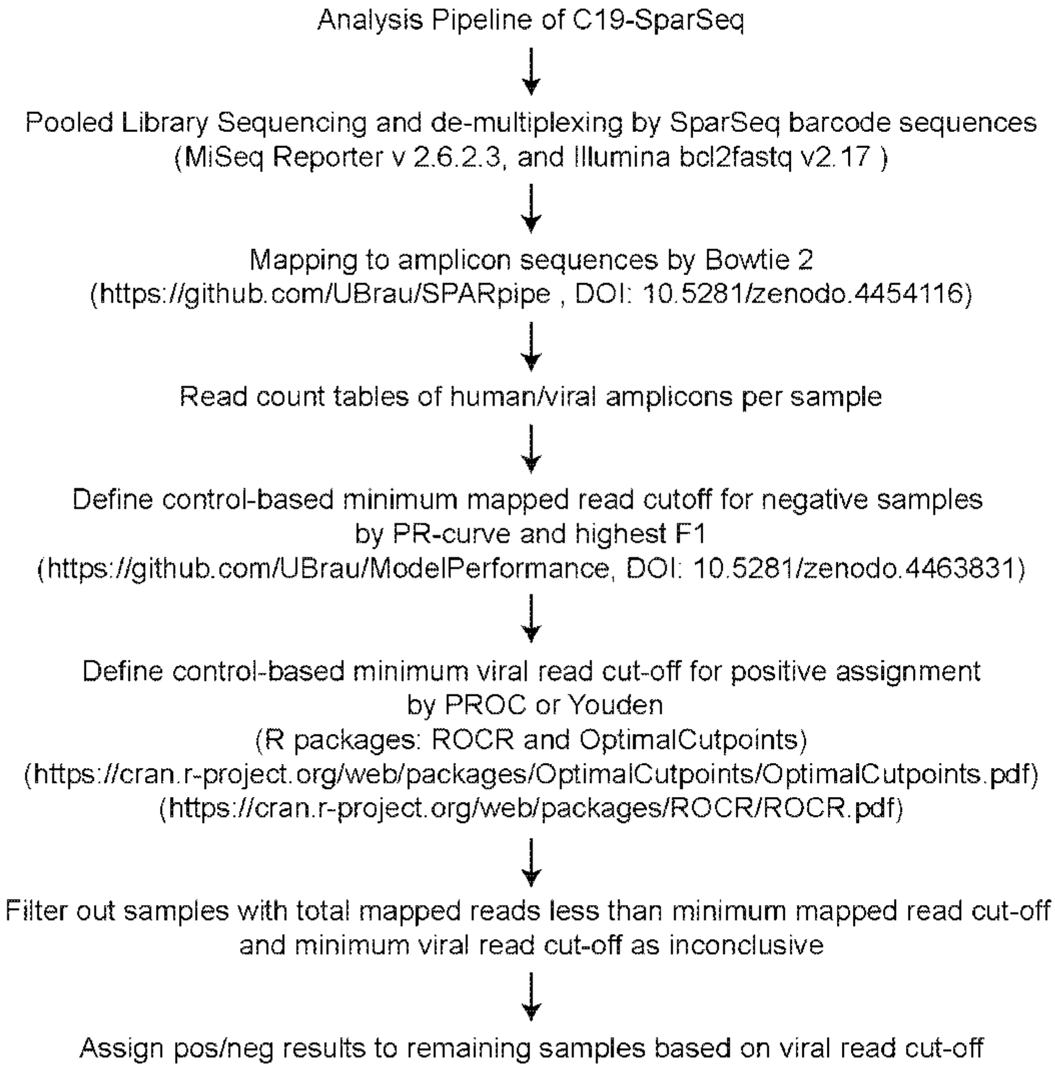

Supplementary Fig. 7: C19-SPAR-Seq analysis and interpretation pipeline. The analysis pipeline is explained step by step and algorithms/tools used are provided in parenthesis for each step.

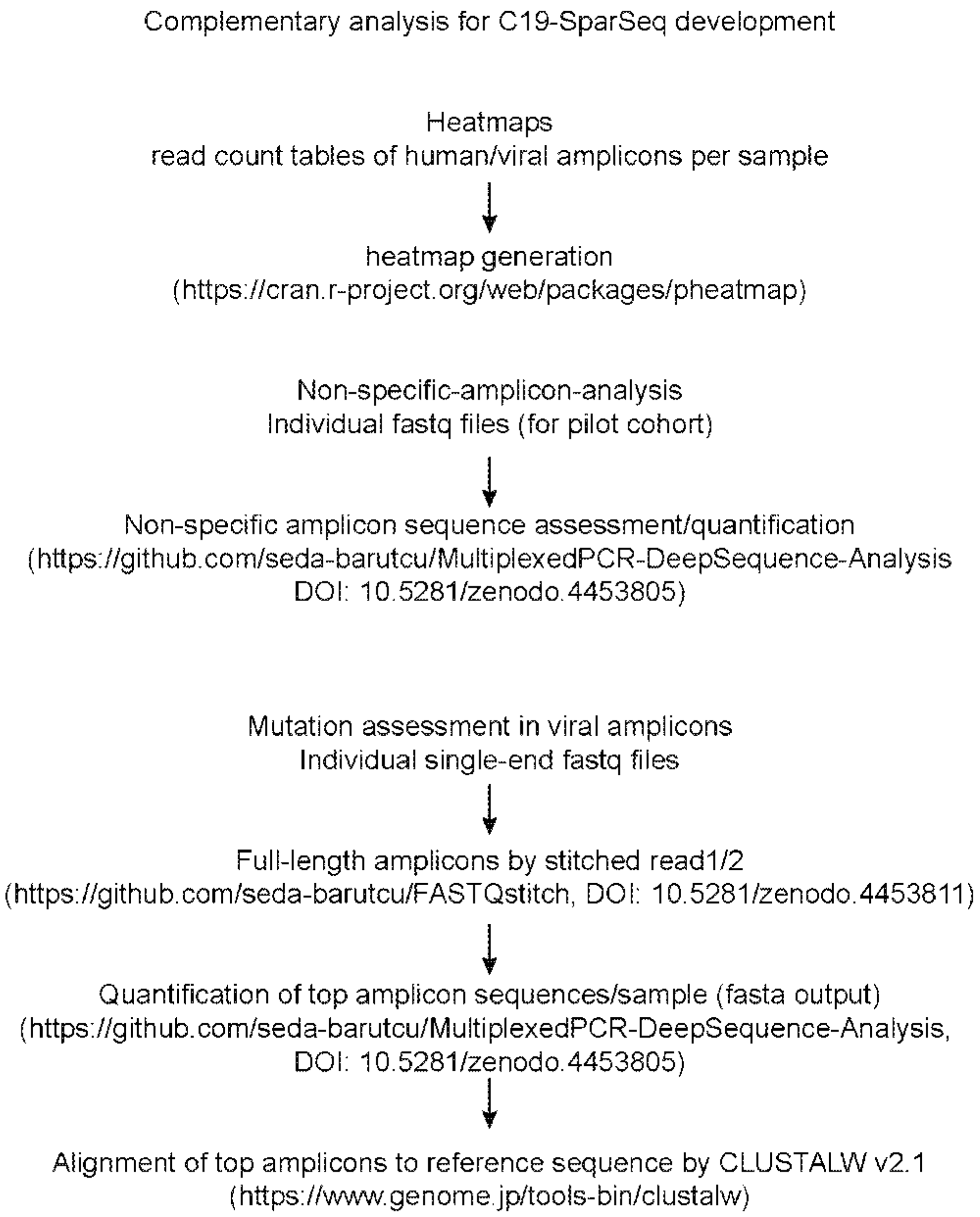

Supplementary Fig. 8: C19-SPAR-Seq complementary analysis. The analysis pipeline is explained step by step and algorithms/tools used are provided in parenthesis for each step.

Name	Sequence
005-PPIB-For	acactctttccctacacgacgctcttccgatctTGCTGCTGCCGGGACCTTC
005-PPIB-Rev	gtgactggagttcagacgtgtgctcttccgatctTTTCCGAAGAGACCAAAGATCACC
013-S-RBD-For	acactctttccctacacgacgctcttccgatctATCAGGCCGGTAGCACACCT
013-S-RBD-Rev	gtgactggagttcagacgtgtgctcttccgatctACTCTGTATGGTTGGTAACCAACAC
014-S-PBS-For	acactctttccctacacgacgctcttccgatctTATGCGCTAGTTATCAGACTCAGAC
014-S-PBS-Rev	gtgactggagttcagacgtgtgctcttccgatctGTAAGCAACTGAATTTTCTGCACCA
006-N-For-v	acactctttccctacacgacgctcttccgatctCCAGGCAGCARTAGGGGAAC
006-N-Rev	gtgactggagttcagacgtgtgctcttccgatctTTGGCCTTTACCAGACATTTTGCTC
008-E-For	acactctttccctacacgacgctcttccgatctAGACAGGTACGTTAATAGTTAATAGCG
008-E-Rev	gtgactggagttcagacgtgtgctcttccgatctCACGTTAACAATATTGCAGCAGTAC
010-RdRP-For-v	acactctttccctacacgacgctcttccgatctTGAGTGARATGGTCATGTGTGGC
010-RdRP-Rev	gtgactggagttcagacgtgtgctcttccgatctGTGACAGCTTGACAAATGTTAAAAAC AC
011-RdRP-For	acactctttccctacacgacgctcttccgatctACCGTTTCTATAGATTAGCTAATGAGT
011-RdRP-Rev	gtgactggagttcagacgtgtgctcttccgatctAAGTGCATTAACATTGGCCGTGAC
023-RdRP-For	acactctttccctacacgacgctcttccgatctGATGCCACAACTGCTTATGC
023-RdRP-Rev	gtgactggagttcagacgtgtgctcttccgatctTTGCGGACATACTTATCGGC
019-ACTB/G-For	acactctttccctacacgacgctcttccgatctTCACCATTGGCAATGAGCGGTTC
019-ACTB/G-Rev	gtgactggagttcagacgtgtgctcttccgatctCCACGTCACACTTCATGATGGAG

For-v: codon optimized primer

Supplementary Table 1: List of SARS-CoV-2 and human primers. Capital letters represent gene specific regions whereas lower-case represent adaptor-specific sequences.

'Seegene': Ct <40		Actual	
		Positive (24)	Negative (73)
Predicted	Positive (22)	22	0
	Negative (75)	2	73

Supplementary Table 2: Confusion matrix of the test development cohort. Predicted patient status is determined by ‘SeeGene’ test (Supplementary Data 4) and Actual patient status is determined by C19-SPAR-Seq results (GSE160032).

'Seegene' : Ct <40	Values
Sensitivity	0.917
Specificity	1
PPV	1
NPV	0.973
Accuracy	0.979

Supplementary Table 3: Statistics of the test development cohort.

Group #	Cohort name	Number of samples	After confirmed diagnosis	Number of positive samples	Number of negative samples
Group 1	Proof of Concept (PoC)	19	19	17	2
Group 2	Test	112	NA	24	88
Group 3	Pilot	378	341	52	289
Group 4	Extended	663	663	98	565

Supplementary Table 4: Group classification.

'BGI' POSITIVE: Ct<37		Actual	
		Positive (98)	Negative (444)
Predicted	Positive (82)	82	0
	Negative (460)	16	444

Supplementary Table 5: Confusion matrix of the extended cohort. Predicted patient status is determined by 'BGI' test (Supplementary Data 6) and Actual patient status is determined by C19-SPAR-Seq results (GSE160034).
