# Supplementary material for: A multiplexed, next generation sequencing platform for high-throughput detection of SARS-CoV-2
Source: Nat Commun. 2021 Mar 3;12:1405. doi: 10.1038/s41467-021-21653-y (PMC7930244; doi:10.1038/s41467-021-21653-y)
Supplement: Supplementary file 2 — Reporting Summary [file 41467_2021_21653_MOESM2_ESM.pdf]

nature research

Corresponding author(s): Dr. Wrana and Dr. Pelletier

Last updated by author(s): Jan 24, 2020

Reporting Summary

Nature Research wishes to improve the reproducibility of the work that we publish. This form provides structure for consistency and transparency in reporting. For further information on Nature Research policies, see our Editorial Policies and the Editorial Policy Checklist.

Statistics

For all statistical analyses, confirm that the following items are present in the figure legend, table legend, main text, or Methods section.

n/a	Confirmed	
☐	☒	The exact sample size (n) for each experimental group/condition, given as a discrete number and unit of measurement
☐	☒	A statement on whether measurements were taken from distinct samples or whether the same sample was measured repeatedly
☐	☒	The statistical test(s) used AND whether they are one- or two-sided<br>*Only common tests should be described solely by name; describe more complex techniques in the Methods section.*
☒	☐	A description of all covariates tested
☒	☐	A description of any assumptions or corrections, such as tests of normality and adjustment for multiple comparisons
☐	☒	A full description of the statistical parameters including central tendency (e.g. means) or other basic estimates (e.g. regression coefficient) AND variation (e.g. standard deviation) or associated estimates of uncertainty (e.g. confidence intervals)
☐	☒	For null hypothesis testing, the test statistic (e.g. F, t, r) with confidence intervals, effect sizes, degrees of freedom and P value noted<br>*Give P values as exact values whenever suitable.*
☒	☐	For Bayesian analysis, information on the choice of priors and Markov chain Monte Carlo settings
☒	☐	For hierarchical and complex designs, identification of the appropriate level for tests and full reporting of outcomes
☐	☒	Estimates of effect sizes (e.g. Cohen's d, Pearson's r), indicating how they were calculated

Our web collection on statistics for biologists contains articles on many of the points above.

Software and code

Policy information about availability of computer code

Data collection	no software were used
Data analysis	Data analysis was performed by MiSeq Reporter v 2.6.2.3, Illumina bcl2fastq v2.17, Prism v 8.1.2, R v 4.0.2, RStudio v1.3.959 ("ROCR", "OptimalCutpoints", "pheatmap" packages), bowtie 2, CLUSTALW v 2.1, and custom scripts available at https://github.com/UBrau/SPARpipe, https://github.com/UBrau/ModelPerformance (DOI: 10.5281/zenodo.4463831), https://github.com/seda-barutcu/MultiplexedPCR-DeepSequence-Analysis (DOI: 10.5281/zenodo.4453805), and https://github.com/seda-barutcu/FASTQstitch (DOI: 10.5281/zenodo.4453811)

For manuscripts utilizing custom algorithms or software that are central to the research but not yet described in published literature, software must be made available to editors and reviewers. We strongly encourage code deposition in a community repository (e.g. GitHub). See the Nature Research guidelines for submitting code & software for further information.

Data

Policy information about availability of data

All manuscripts must include a data availability statement. This statement should provide the following information, where applicable:

- Accession codes, unique identifiers, or web links for publicly available datasets
- A list of figures that have associated raw data
- A description of any restrictions on data availability

-Severe acute respiratory syndrome coronavirus 2 isolate Wuhan-Hu-1, complete genome : NCBI sequence ID: NC_045512
-Figure1 raw data, PoC cohort: GEO accession number GSE160031
-Figure2 and Supplementary Figure2 raw data, Test cohort: GEO accession number GSE160032
-Figure3 and Supplementary Figure3-4 raw data, Pilot cohort: GEO accession number GSE160033
-Figure4 and Supplementary Figure5-6 raw data, Extended cohort: GEO accession number GSE160034

-All data-sets and corresponding FASTQ files can be found under accession number: GSE160036

Field-specific reporting

Please select the one below that is the best fit for your research. If you are not sure, read the appropriate sections before making your selection.

- [x] Life sciences
- [ ] Behavioural & social sciences
- [ ] Ecological, evolutionary & environmental sciences

For a reference copy of the document with all sections, see nature.com/documents/nr-reporting-summary-flat.pdf

Life sciences study design

All studies must disclose on these points even when the disclosure is negative.

Sample size	Sample sizes for each group were determined based on availability of archived material. Each cohort increased in sample size as we continued to optimize our method for high-throughput detection of SARS-CoV-2, and a proportionate number of negative samples were included to recapitulate positive infection rates within the population.
Data exclusions	39 putative positive samples were excluded because we were unable to confirm positivity.
Replication	Clinical diagnosis of samples were validated by benchmarking with both Beijing Genomics Institute qRT-PCR and SeeGene Allplex qRT-PCR assays, as approved by CDC guidelines.
Randomization	Samples were not randomized in this study. Samples were allocated as either negative- or positive-SARS-CoV-2 based on both time of collection (pre- and post-SARS-CoV-2 retrieval) and clinical diagnosis by standard procedures.
Blinding	Experiments were not blinded given that we already knew clinical diagnostic result of each sample.

Reporting for specific materials, systems and methods

We require information from authors about some types of materials, experimental systems and methods used in many studies. Here, indicate whether each material, system or method listed is relevant to your study. If you are not sure if a list item applies to your research, read the appropriate section before selecting a response.

Materials & experimental systems

n/a	Involved in the study	
x		Antibodies
	x	Eukaryotic cell lines
x		Palaeontology and archaeology
x		Animals and other organisms
	x	Human research participants
x		Clinical data
x		Dual use research of concern

Methods

n/a	Involved in the study	
x		ChIP-seq
x		Flow cytometry
x		MRI-based neuroimaging

Eukaryotic cell lines

Policy information about cell lines

Cell line source(s)	ATCC human embryonic kidney 293 cells, and contains the SV40 T-antigen (HEK293T)
Authentication	Cell line was not authenticated
Mycoplasma contamination	Regularly perform mycoplasma testing in our lab cultures. This cell line was tested negative for mycoplasma.
Commonly misidentified lines (See ICLAC register)	No commonly misidentified cell lines were used in this study.

Human research participants

Policy information about studies involving human research participants

Population characteristics	No age or gender criteria for sample collection (Details in the methods). Sample collection methods varied across patients, including nasal swabs, nasopharyngeal swabs, broncho-alveolar lavages and bronchial washes.
Recruitment	Patients presented symptoms of respiratory pathologies which warranted pathogen testing.
Ethics oversight	Mount Sinai Hospital Research Ethics Board (Study #20-0078-E)

Note that full information on the approval of the study protocol must also be provided in the manuscript.
