# Supplementary material for: A multiplexed, next generation sequencing platform for high-throughput detection of SARS-CoV-2
Source: Nat Commun. 2021 Mar 3;12:1405. doi: 10.1038/s41467-021-21653-y (PMC7930244; doi:10.1038/s41467-021-21653-y)
Supplement: Supplementary file 10 — Description of Additional Supplementary Files [file 41467_2021_21653_MOESM10_ESM.pdf]

Title: Supplementary Data 1.
Description: List of SARS-CoV-2 and human primers. Primer sequences, name of the targeted regions, size of amplicons after multiplex and barcode PCR are indicated.

Title: Supplementary Data 2.
Description: Itemized cost of C19-SPAR-Seq per sample

Title: Supplementary Data 3.
Description: Description of the proof-of-concept cohort for C19- SPAR-Seq detection of SARS-CoV-2. Barcodes ID, sample identification (ID), date of retrieval, collection method, diagnostic laboratory status, and 'BGI' qRT-PCR results are indicated. These patient samples were used to develop C19-SPAR-Seq detection of SARS-CoV-2 (PoC cohort) (Fig. 1).

Title: Supplementary Data 4.
Description: Description of test development cohort. Barcodes ID, sample identification (ID), date of retrieval, collection method, diagnostic laboratory qRT-PCR results ('Seegene') are indicated (n = 112). These patient samples were used to establish SARS-CoV-2 clinical status assignment using diagnostic laboratory qRT-PCR results ('Seegene') and to test C19-SPAR-Seq detection of SARS-CoV-2 (Fig. 2,3).

Title: Supplementary Data 5.
Description: Description of the pilot cohort. Barcodes ID, sample identification (ID), date of retrieval, collection method, diagnostic laboratory qRT-PCR results ('Seegene'), 'BGI' qRT-PCR results are indicated. Filtered archival samples are indicated. (Extended data Fig. 3,4).

Title: Supplementary Data 6.
Description: Description of the extended cohort. Barcodes ID, sample identification (ID), date of retrieval, collection method, diagnostic laboratory qRT-PCR results ('Seegene'), and 'BGI' qRT-PCR results are indicated (Fig. 4).
