# Supplementary material for: A multiplexed, next generation sequencing platform for high-throughput detection of SARS-CoV-2
Source: Nat Commun. 2021 Mar 3;12:1405. doi: 10.1038/s41467-021-21653-y (PMC7930244; doi:10.1038/s41467-021-21653-y)
Supplement: Supplementary file 11 — Source Data [file 41467_2021_21653_MOESM11_ESM.zip › SourceData/SourceData2_SupplementaryFig4c.pdf]

Sample: Spar_Seq_run3_PoolLib_After_Clean_Apr222020
Well Location: A8
Created: Wednesday, April 22, 2020 4:37:44 PM

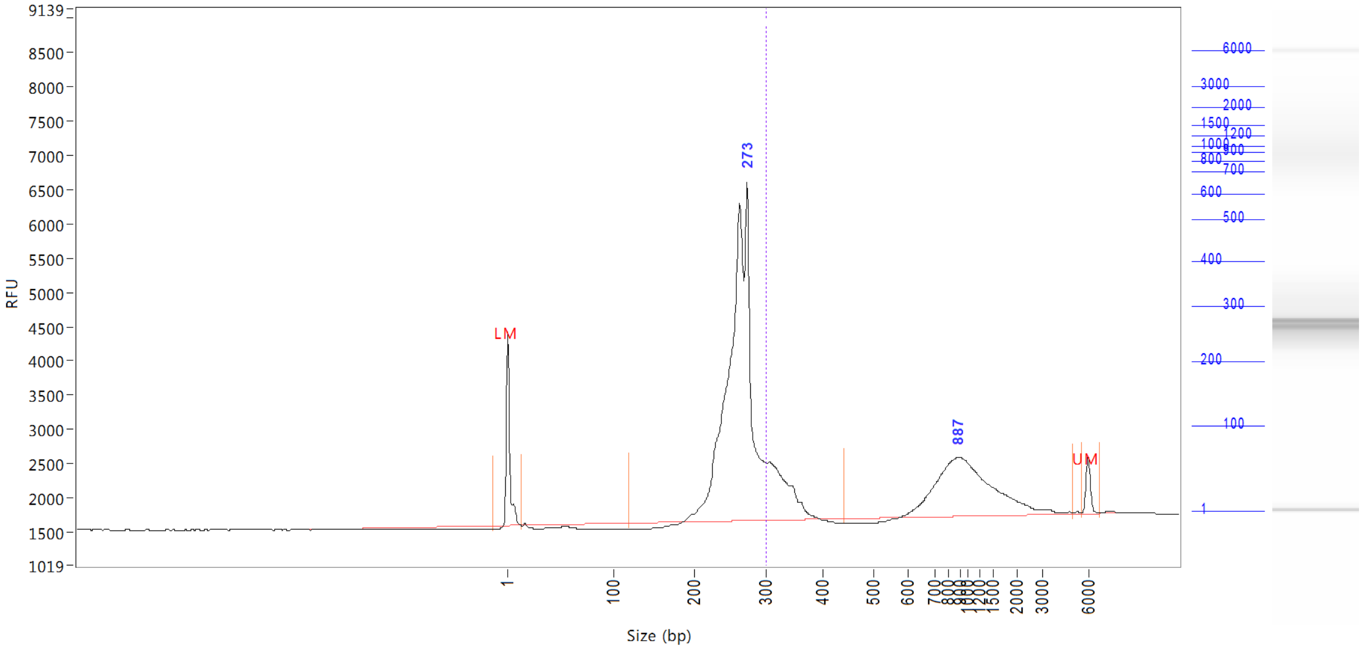

Peak	Size (bp)	Conc. (ng/uL)	From (bp)	To (bp)	Avg. Size (bp)	CV%	RFU	Corr. Peak Area
1	1 (LM)	0.6477	0	14	1	235.95	2819	25.095
2	273	66.7149	118	441	268	11.79	4934	215.401
3	887	19.7556	441	5033	1153	51.17	865	63.785
4	6000 (UM)	0.1111	5613	6816	6005	2.87	827	4.304

TIC:	86.4705	ng/uL
TIM:	437.6443	nmole/L
Total Conc.:	86.5569	ng/uL

DQN:	3.4
Threshold:	300

Sample Peak Width (sec): 50 Sample Min Peak Height: 25 Sample Baseline V to V?: Y Sample Baseline V to V pts: 3
Sample Filter: Binomial # of Pts for Filter: 3 Sample Start Region (min): 0 Sample End Region (min): 25
Manual Baseline Start (min): 5 Manual Baseline End (min): 24
Marker Peak Width (sec): 3 Marker Min Peak Height: 200 Marker Baseline V to V?: Y Marker Baseline V to V pts: 3
Lower Marker Selection: First Peak > 200 RFU Upper Marker Selection: Last Peak > 200 RFU
Ladder Size (bp)1, 100, 200, 300, 400, 500, 600, 700, 800, 900, 1000, 1200, 1500, 2000, 3000, 6000
Quantification Using: Ladder Final Concentration (ng/uL): 2.0830 Dilution Factor: 12.0
Size Threshold (b.p.): 300

 Copyright 2015 Advanced Analytical Technologies, Inc.

Sample: SparSeq_Run10_Pooled_After_beads
Well Location: G2
Created: Tuesday, June 23, 2020 12:30:51 PM

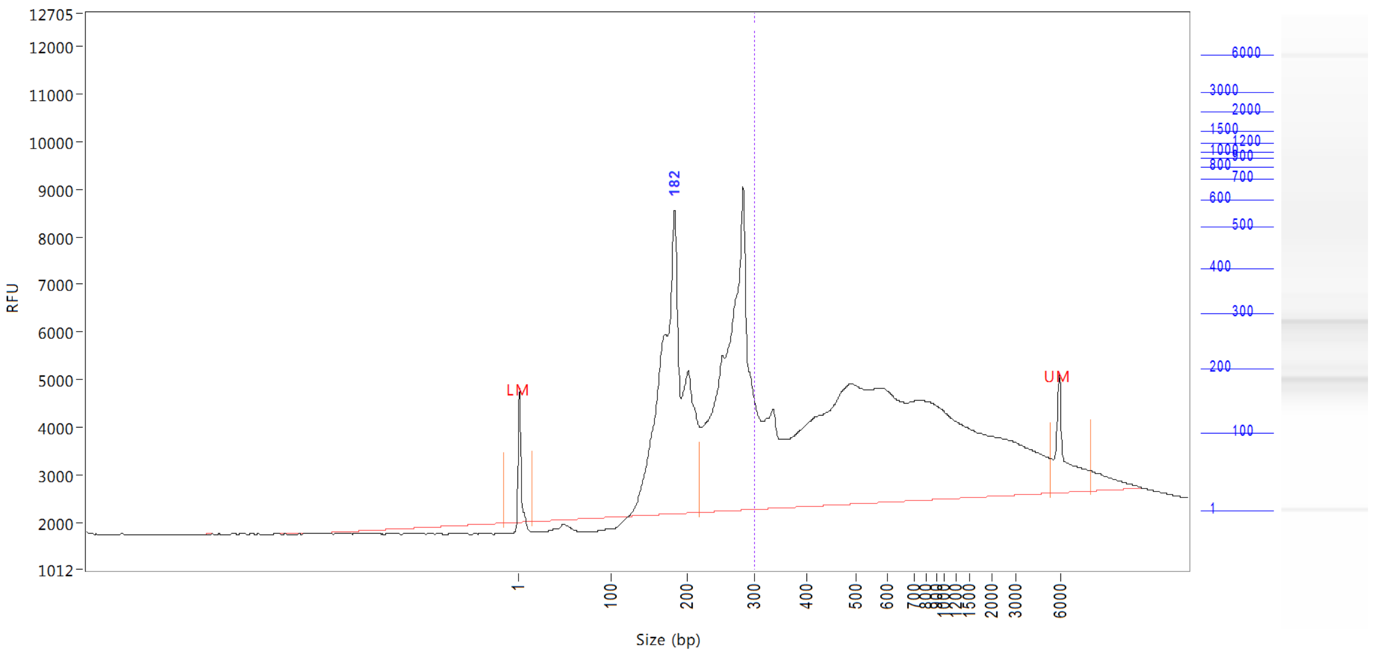

Peak	Size (bp)	Conc. (ng/uL)	From (bp)	To (bp)	Avg. Size (bp)	CV%	RFU	Corr. Peak Area
1	1 (LM)	0.0235	0	15	1	309.49	2823	20.125
2	182	4.0118	15	219	179	10.93	6381	286.093
3	6000 (UM)	0.0372	5468	8157	6491	11.48	2494	31.796

TIC:	4.0118	ng/uL
TIM:	36.8453	nmole/L
Total Conc.:	16.9948	ng/uL

DQN:	5.4
Threshold:	300

Sample Peak Width (sec): 50 Sample Min Peak Height: 25 Sample Baseline V to V?: Y Sample Baseline V to V pts: 3
Sample Filter: Binomial # of Pts for Filter: 3 Sample Start Region (min): 0 Sample End Region (min): 25
Manual Baseline Start (min): 5 Manual Baseline End (min): 24
Marker Peak Width (sec): 3 Marker Min Peak Height: 200 Marker Baseline V to V?: Y Marker Baseline V to V pts: 3
Lower Marker Selection: First Peak > 200 RFU Upper Marker Selection: Last Peak > 200 RFU
Ladder Size (bp)1, 100, 200, 300, 400, 500, 600, 700, 800, 900, 1000, 1200, 1500, 2000, 3000, 6000
Quantification Using: Ladder Final Concentration (ng/uL): 0.0830 Dilution Factor: 12.0
Size Threshold (b.p.): 300

 Copyright 2015 Advanced Analytical Technologies, Inc.

Sample: SparSeq_Run11_457_Bead_Cleanup_June262020
Well Location: C2
Created: Friday, June 26, 2020 4:29:28 PM

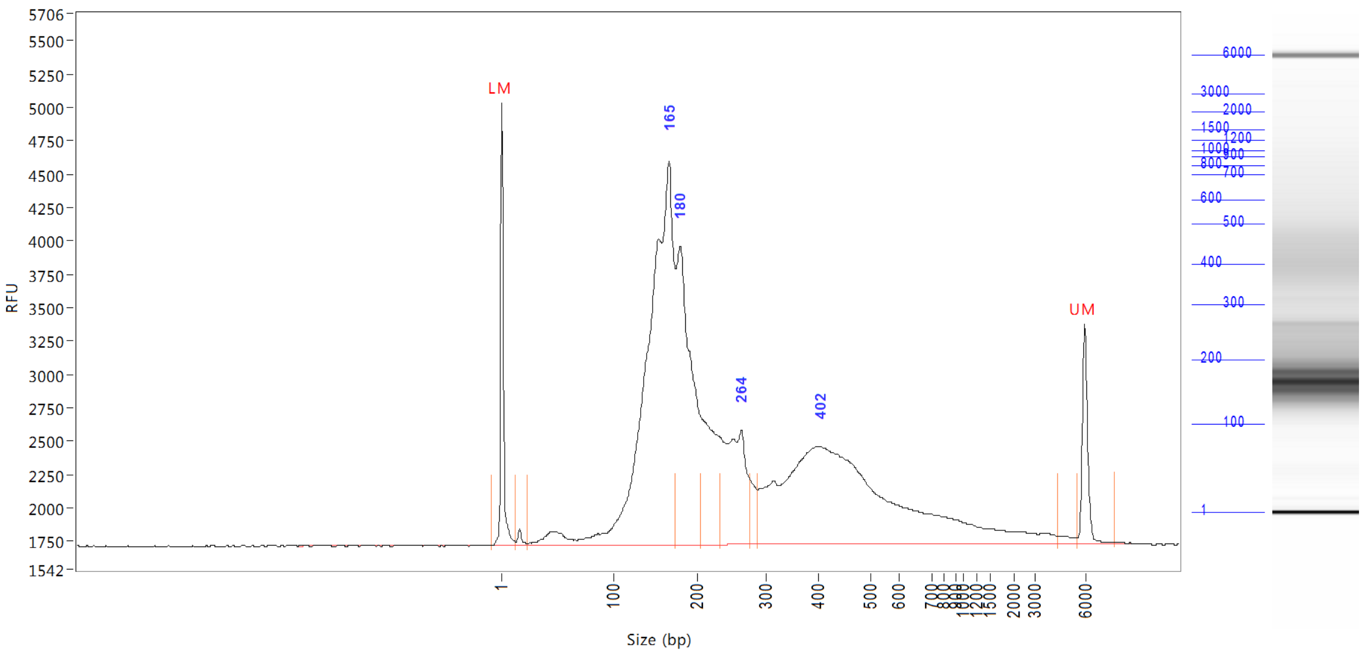

Peak	Size (bp)	Conc. (ng/uL)	From (bp)	To (bp)	Avg. Size (bp)	CV%	RFU	Corr. Peak Area
1	1 (LM)	0.2434	0	14	1	381.63	3319	28.110
2	165	29.4994	23	287	175	26.34	2877	283.926
3	180	6.9346	174	205	186	4.79	2230	66.745
4	264	3.4911	233	275	253	4.70	859	33.601
5	402	13.1577	287	4410	576	87.61	735	126.640
6	6000 (UM)	0.0854	5546	7804	6004	3.76	1641	9.860

TIC:	53.0828	ng/uL
TIM:	398.6932	nmole/L
Total Conc.:	42.8340	ng/uL

```
Sample Peak Width (sec): 50   Sample Min Peak Height: 25    Sample Baseline V to V?: Y    Sample Baseline V to V pts: 3
Sample Filter: Binomial       # of Pts for Filter: 3        Sample Start Region (min): 0  Sample End Region (min): 25
Manual Baseline Start (min): 5     Manual Baseline End (min): 24
Marker Peak Width (sec): 3    Marker Min Peak Height: 200   Marker Baseline V to V?: Y    Marker Baseline V to V pts: 3
Lower Marker Selection: First Peak > 200 RFU                Upper Marker Selection: Last Peak > 200 RFU
Ladder Size (bp)1, 100, 200, 300, 400, 500, 600, 700, 800, 900, 1000, 1200, 1500, 2000, 3000, 6000
Quantification Using: Ladder          Final Concentration (ng/uL): 2.0830          Dilution Factor: 12.0
```


Copyright 2015 Advanced Analytical Technologies, Inc.
