# Supplementary material for: A multiplexed, next generation sequencing platform for high-throughput detection of SARS-CoV-2
Source: Nat Commun. 2021 Mar 3;12:1405. doi: 10.1038/s41467-021-21653-y (PMC7930244; doi:10.1038/s41467-021-21653-y)
Supplement: Supplementary file 11 — Source Data [file 41467_2021_21653_MOESM11_ESM.zip › SourceData/SourceData3_SupplementaryFig5a.pdf]

Supplementary Fig. 5a Fragment analyzer profil of the extended cohort library

Sample: SparSeq Run 20 After 1X Beads Cleanup
Well Location: C2
Created: Tuesday, September 1, 2020 12:20:07 PM

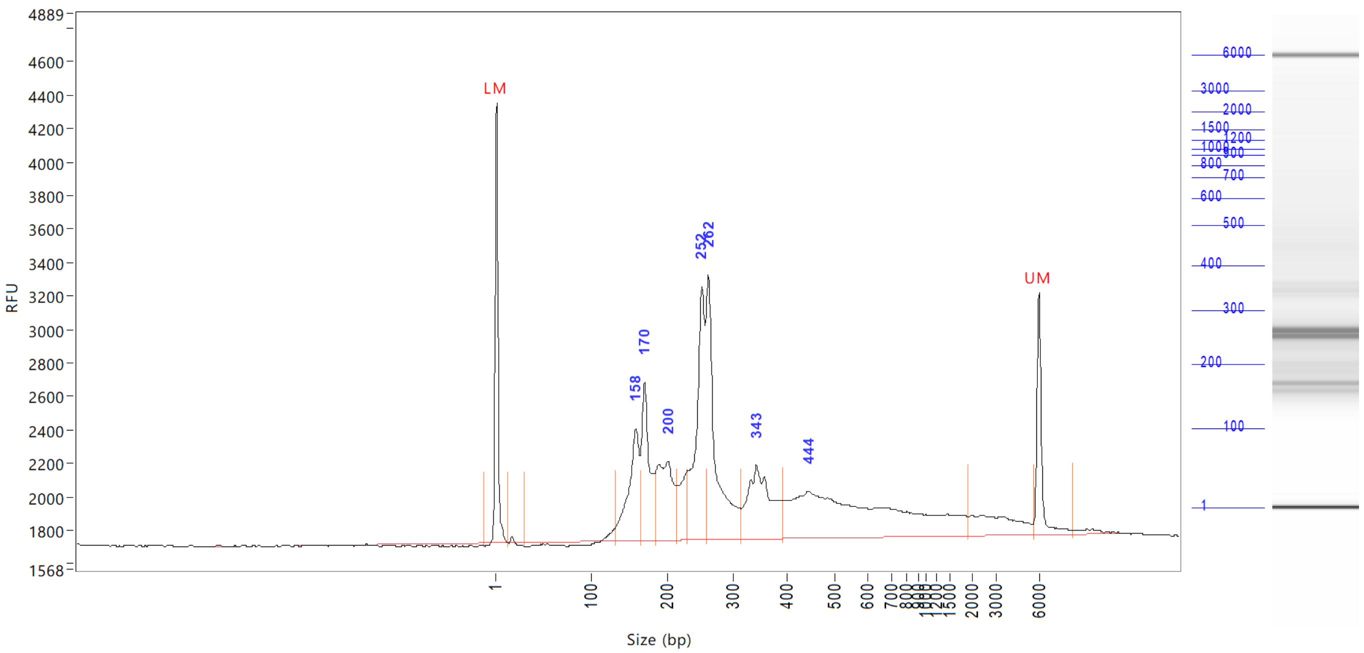

Peak	Size (bp)	Conc. (ng/uL)	From (bp)	To (bp)	Avg. Size (bp)	CV%	RFU	Corr. Peak Area
1	1 (LM)	0.5101	0	14	0	593.32	2631	21.230
2	158	4.8996	130	166	153	5.97	673	16.994
3	170	13.5133	31	214	173	12.47	948	46.869
4	200	6.1963	184	230	205	6.73	471	21.491
5	252	7.8044	231	260	249	3.03	1508	27.069
6	262	16.6795	214	315	257	8.06	1579	57.851
7	343	5.2342	315	393	352	6.01	441	18.154
8	444	12.2609	393	1947	687	51.68	283	42.525
9	6000 (UM)	0.1972	5586	8300	6112	8.07	1447	8.208

TIC:	66.5883	ng/uL
TIM:	442.8651	nmole/L
Total Conc.:	49.5135	ng/uL

```
Sample Peak Width (sec): 50   Sample Min Peak Height: 25    Sample Baseline V to V?: Y    Sample Baseline V to V pts: 3
Sample Filter: Binomial       # of Pts for Filter: 3        Sample Start Region (min): 0  Sample End Region (min): 25
Manual Baseline Start (min): 5     Manual Baseline End (min): 24
Marker Peak Width (sec): 3    Marker Min Peak Height: 200   Marker Baseline V to V?: Y    Marker Baseline V to V pts: 3
Lower Marker Selection: First Peak > 200 RFU                Upper Marker Selection: Last Peak > 200 RFU
Ladder Size (bp)1, 100, 200, 300, 400, 500, 600, 700, 800, 900, 1000, 1200, 1500, 2000, 3000, 6000
Quantification Using: Ladder          Final Concentration (ng/uL): 2.0830          Dilution Factor: 12.0
```

 Copyright 2015 Advanced Analytical Technologies, Inc.
